# A Case of Triplet Therapy Showing Remarkable Efficacy for Multiorgan Metastatic Recurrence After Radical Prostatectomy in Prostate Cancer

**DOI:** 10.1002/iju5.70082

**Published:** 2025-08-18

**Authors:** Shuhei Kusano, Shohei Tobu, Minika Yukimoto, Yukako Yamaguchi, Yuka Kakinoki, Akihiro Maeda, Maki Kawasaki, Hiroaki Kakinoki, Mitsuru Noguchi

**Affiliations:** ^1^ Department of Urology, Faculty of Medicine, Saga University, Saga, Japan

**Keywords:** degarelix, docetaxel, mCSPC, triplet therapy darolutamide

## Abstract

**Introduction:**

We report a case in which triplet therapy demonstrated efficacy for multiple metastatic recurrences following radical prostatectomy.

**Case Presentation:**

A 70‐year‐old man with relapsed metastatic castration‐sensitive prostate cancer (mCSPC) following radical prostatectomy (Gleason 9, pT3bN1M0) presented with rectal involvement and extensive lymph node and bone metastases, as evidenced by a markedly elevated PSA level of 59.57 ng/mL. He received triplet therapy consisting of androgen deprivation therapy (ADT) with degarelix, darolutamide (1200 mg/day), and docetaxel (70 mg/m^2^). This combination led to a complete PSA response, dropping below the detection limit (< 0.006 ng/mL). At 24 months post‐treatment, the patient remained in a stable condition without any signs of PSA recurrence.

**Conclusion:**

This case highlights the potential of triplet therapy as a highly effective treatment strategy for high‐risk mCSPC patients who experience recurrence after initial local therapy.

**Summary**

- A 70‐year‐old man with metastatic castration‐sensitive prostate cancer post‐radical prostatectomy, exhibiting extensive metastases and high PSA, achieved complete PSA and radiological remission with triplet therapy (degarelix, darolutamide, docetaxel). This case highlights triplet therapy's efficacy for recurrent, high‐risk mCSPC, warranting further research into patient selection and long‐term outcomes.

## 1 Introduction

Androgen deprivation therapy (ADT) is the standard treatment for metastatic castration‐sensitive prostate cancer (mCSPC). In recent years, triplet therapy, which combines novel androgen receptor‐targeted agents (ARTA) with docetaxel (DOC), has garnered attention. In Japan, based on evidence from trials such as ARASENS, aggressive systemic therapy from the initial treatment stage is now recommended. We report a unique and valuable case in which a patient with recurrent mCSPC who developed multiple metastases after radical prostatectomy was treated with ADT combined with darolutamide and docetaxel, resulting in a significant decrease in PSA and complete radiographic remission.

## 2 Case Presentation

The patient was a 70‐year‐old man who underwent radical prostatectomy at another hospital at 62 years of age. The pathological findings were adenocarcinoma, Gleason score of 4 + 5 = 9, and pT3bN1M0. At 64 years of age, he was referred to our hospital following relocation. His PSA level was low (< 0.006 ng/mL) and follow‐up was performed at a nearby clinic. However, he failed to attend his regular follow‐up appointments.

**FIGURE 1:**
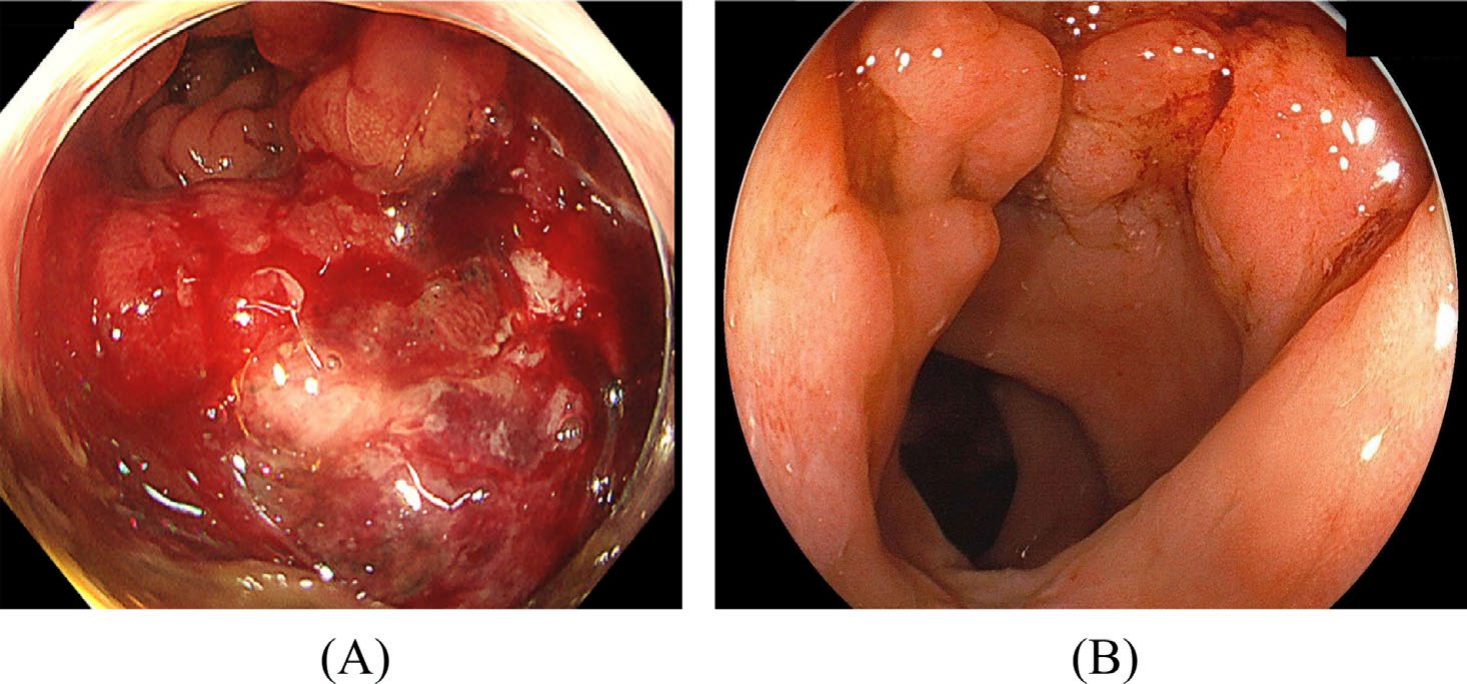
Colonoscopy findings: (A) Pre‐treatment findings: A circumferentially elevated lesion was observed in the rectum. (B) Post‐treatment findings: The raised lesions of the rectal mucosa demonstrated improvement.

**FIGURE 2:**
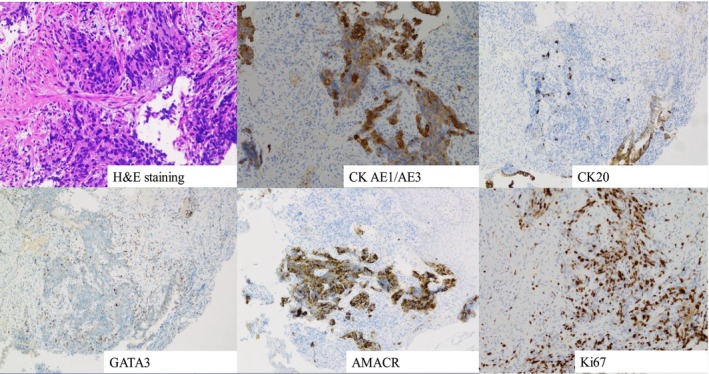
Tumor biopsy pathology. H&E staining and various immunohistochemical stains.

At 70 years of age, he was presented to the nearby clinic with chief complaints of weight loss and hematochezia and was referred to our hospital's Department of Gastroenterology with suspected rectal cancer. Lower gastrointestinal endoscopy revealed a tumorous lesion extending from the anal canal to the rectum (Figure 1A). Immunohistochemical staining of a biopsy specimen showed the following results: CK AE1/AE3 positive, AMACR positive, CK20 negative, and GATA3 negative (Figure 2). Considering his past medical history, recurrent prostate cancer with direct rectal invasion was strongly suspected. A repeat PSA test revealed a markedly elevated PSA level of 59.57 ng/mL.

## 3 Physical Examination and Laboratory Findings

The patient's height and weight were 171.2 cm and 55.4 kg, respectively. A digital rectal examination revealed an induration near the anus.

A peripheral blood test showed the following results: WBC 11.2 × 10^3^/μL (neutrophils, 72.3%), Hb 15.2 g/dL, and PLT 24.9 × 10^4^/μL. Biochemical tests revealed the following: total protein 7.6 g/dL, albumin 3.8 g/dL, total bilirubin 1.1 mg/dL, AST 17 U/L, ALT 22 U/L, LDH 169 U/L, γ‐GTP 18 U/L, amylase 57 U/L, glucose 106 mg/dL, HbA1c 6.9%, BUN 13.2 mg/dL, creatinine 0.63 mg/dL, sodium 140 mmol/L, potassium 4.8 mmol/L, chloride 101 mmol/L, calcium 9.2 mg/dL, and CRP 4.96 mg/dL. PSA was elevated at 59.5 ng/mL. A urinalysis revealed pH 7.0, specific gravity 1.030, urine glucose (3+), urine protein (negative), urine ketones (negative), urine occult blood (negative), nitrite (negative), and leukocytes (negative).

PET‐CT revealed tumorous thickening of the rectal wall from the rectosigmoid junction to the anal canal, with heterogeneous FDG uptake. Multiple enlarged lymph nodes with high FDG uptake were also observed in the sigmoid mesentery, para‐aortic region extending to the retrocrural space, around the thoracic aorta, and left supraclavicular fossa. Furthermore, mild FDG uptake was noted on the left side of the rectum near the levator ani muscle, suggestive of lymph node metastasis or peritoneal dissemination.

**FIGURE 3:**
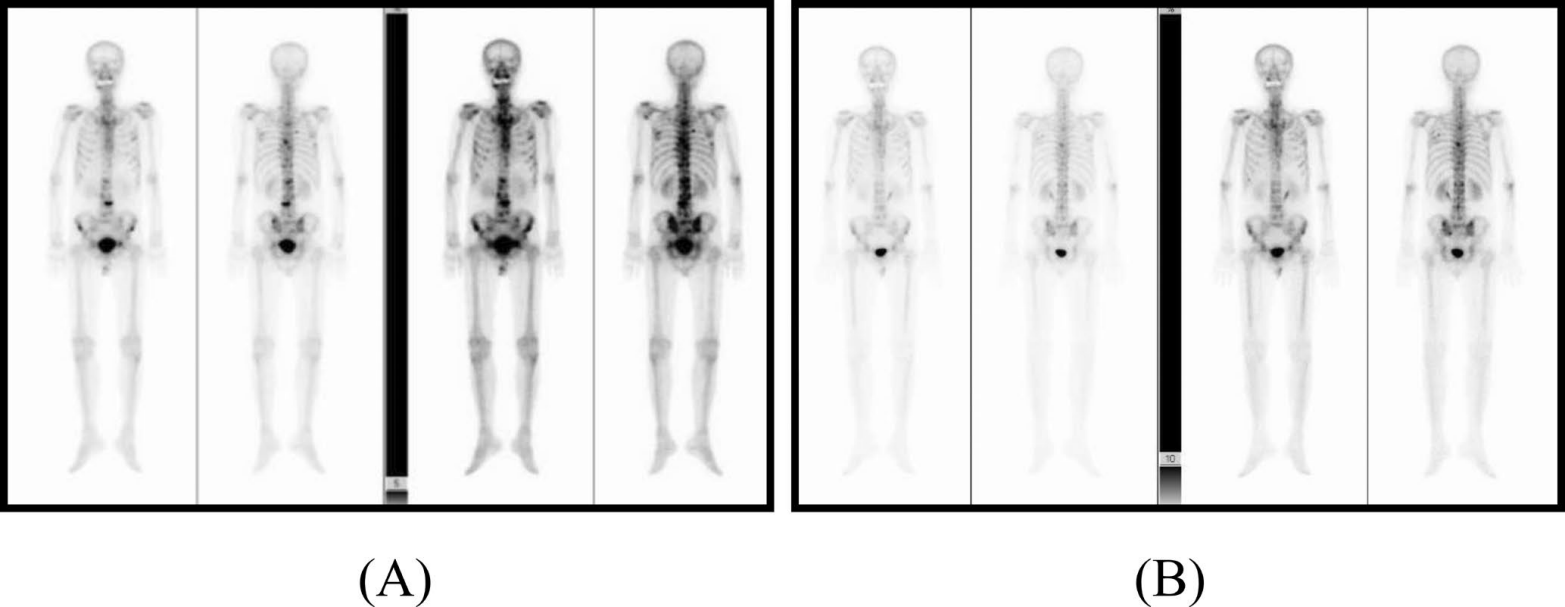
(A) Pre‐treatment findings: Bone scintigraphy showed multiple mild areas of uptake in the right 7th rib, spine, and pelvic bones, with scattered areas of increased bone marrow activity in both femurs and faint uptake. (B) Post‐treatment findings: Overall improvement in radiotracer uptake was observed after triplet therapy.

Bone scintigraphy showed multiple mild areas of uptake in the right 7th rib, spine, and pelvic bones, with scattered areas of increased bone marrow activity in both femurs and faint uptake (Figure 3A).

These findings strongly suggested advanced prostate cancer with multiple bone and lymph node metastases.

## 4 Treatment and Clinical Course

The patient was diagnosed with mCSPC after radical prostatectomy and treated with triplet therapy consisting of degarelix acetate, darolutamide (1200 mg/day), and DOC (70 mg/m^2^). Pegfilgrastim (3.6 mg) was administered on day 3 of each chemotherapy cycle to prevent neutropenia. One month after starting the treatment, his PSA level decreased to 2.82 ng/mL. Grade 3 ALT elevation was observed before the second course of docetaxel, which was temporarily discontinued for 1 month. It was subsequently restarted at 75% of the original dose, and the patient completed six courses of docetaxel without further adverse events. As another adverse event, the patient experienced grade 2 alopecia.

**FIGURE 4:**
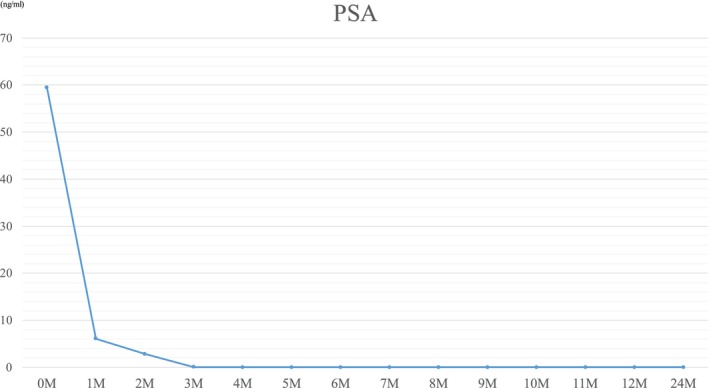
Progression of PSA levels during prostate cancer treatment.

At the end of treatment, his PSA level was < 0.006 ng/mL, and bone scintigraphy showed improvement in radiotracer uptake at the sites of bone metastases (Figure 3B). Colonoscopy also demonstrated the disappearance of the tumorous lesion and smoothing of the mucosa (Figure 1B). Currently, 24 months after the initiation of treatment, the patient is stable without PSA recurrence and continues to receive darolutamide and ADT (Figure 4).

## 5 Discussion

ADT is the standard treatment for mCSPC. However, treatment strategies combining DOC or ARTA with ADT to improve survival outcomes have been recommended in recent years.

The CHAARTED [1] and STAMPEDE [2] trials demonstrated that combining ADT with DOC significantly prolonged overall survival (OS) relative to ADT alone. Furthermore, the efficacy of combining ARTAs, such as abiraterone or enzalutamide, with ADT has been confirmed in several studies [3, 4]. Based on this evidence, the European Association of Urology (EAU) guidelines consider the combination of ADT with DOC or ADT with ARTA as a standard treatment to intensify therapy in mCSPC [5]. Given the extensive disease burden observed in this case—including direct rectal invasion, multiple enlarged lymph nodes, and bone metastases—the patient fulfilled the criteria for high‐volume disease as defined in clinical trials such as CHAARTED and ARASENS. In this context, we selected triplet therapy over doublet therapy based on current guidelines and the proven survival benefit in patients with high‐volume metastatic disease. This approach was further supported by the patient's good performance status and ability to tolerate chemotherapy.

More recently, triplet therapy, which combines ADT with DOC and ARTA, has garnered attention, and its efficacy has been demonstrated in large‐scale trials. The ARASENS trial [6] showed that the triplet therapy group, which combined ADT and DOC with darolutamide, significantly prolonged OS relative to the ADT, DOC, and placebo groups, reducing the risk of death by 32% (HR 0.68, 95% CI: 0.57–0.80, *p* < 0.0001). The superiority of triplet therapy was also confirmed by secondary endpoints.

Recent network meta‐analyses have shown that ADT, DOC, and ARTA triplet therapy demonstrate superior treatment outcomes in comparison to ADT and DOC [7]. However, there are no direct comparison trials of triplet therapy versus ADT and ARTA; it cannot be definitively concluded that triplet therapy is superior to dual therapy. Treatment selection should comprehensively consider the extent of metastasis, patient characteristics, and tolerance to side effects.

Notably, most clinical trials supporting triplet therapy, including the ARASENS study, primarily enrolled patients with *de novo* metastatic disease. The applicability of these findings to patients with *metachronous* metastases—those who relapse after definitive local therapy—remains less clearly defined. Our case falls into this latter category, which may represent a biologically and clinically distinct subgroup. *Metachronous* mCSPC is often associated with more indolent disease biology and a potentially more favorable prognosis compared to *de novo* cases. However, in this patient, the extent of metastatic spread—combined with rectal invasion—suggested an aggressive recurrence pattern. Thus, despite the absence of direct evidence for triplet therapy in *metachronous* settings, we opted for this intensified approach, anticipating that aggressive biology would benefit from upfront multimodal systemic control.

## 6 Conclusion

We report a case of mCSPC with distant metastases following radical prostatectomy, in which ADT, darolutamide, and docetaxel triplet therapy achieved complete PSA remission. Triplet therapy is a highly promising treatment option for high‐risk cases, and further investigation of its indications and long‐term outcomes is required.

## Consent

The consent was obtained from the patient.

## Conflicts of Interest

The authors declare no conflicts of interest.

## Data Availability

The datasets analyzed during this study are available from the corresponding author on reasonable request.
